# ﻿Morpho-phylogenetic evidence reveals new species and records of Beltraniaceae (Amphisphaeriales, Sordariomycetes) from southern China

**DOI:** 10.3897/mycokeys.123.160374

**Published:** 2025-09-24

**Authors:** Qiyun Liu, Duhua Li, Zhaoxue Zhang, Yuefeng Geng, Zhuang Li, Xiuguo Zhang, Jiwen Xia

**Affiliations:** 1 College of Agriculture and Forestry, Linyi University, Linyi, Shandong, 276000, China Linyi University Linyi China; 2 College of Plant Protection, Shandong Agricultural University, Taian, Shandong, 271018, China Shandong Agricultural University Taian China; 3 Liaocheng Agricultural Technology Extension Service Center, Liaocheng, Shandong, 252000, China Liaocheng Agricultural Technology Extension Service Center Liaocheng China

**Keywords:** *
Beltrania
*, *
Beltraniella
*, morphology, new taxa, phylogeny

## Abstract

Beltraniaceae comprises mainly saprophytic fungi that occur on various substrates in tropical and subtropical regions worldwide. In our ongoing survey of terrestrial plant fungi in southern China, we collected Beltraniaceae isolates from Guizhou, Hainan, and Yunnan provinces. Maximum likelihood and Bayesian inference analyses based on two genetic loci (ITS and LSU) were employed to determine the taxonomic position of the species. We confirmed that they represent two new species (*Beltrania veri***sp. nov.** and *Beltraniella danzhouensis***sp. nov.**), three newly recorded species (*Beltrania sinensis*, *Beltraniella fertilis*, and *Beltraniella jiangxiensis*), and one known species (*Beltraniella jianfengensis*). We provide detailed descriptions and micrographs of these species and compare them with other Beltraniaceae species.

## ﻿Introduction

Nannizzi first proposed the family Beltraniaceae in 1934 to accommodate the type genus *Beltrania* Penz. and some similar genera (Lin et al. 2017a). Beltraniaceae currently comprises *Beltrania* and 10 allied genera, including *Anabeltraniomyces* R.F. Castañeda, Mardones, P.M. Kirk & Gusmão, *Beltraniella* Subram., *Beltraniopsis* Bat. & J.L. Bezerra, *Hemibeltrania* Piroz., *Parabeltrania* Rambelli ex R.F. Castañeda, Gusmão & P.M. Kirk, *Parapleurotheciopsis* P.M. Kirk, *Porobeltraniella* Gusmão, *Pseudobeltrania* Henn., *Pseudosubramaniomyces* Crous, and *Subsessila* C.G. Lin & K.D. Hyde (Hyde et al. 2024). Members of Beltraniaceae are characterized by biconic, lageniform to navicular conidia that may have a hyaline band and swollen separating cells, while their conidiophores either develop independently or arise from setal basal cells, which may show radial lobing (Lin et al. 2017b; Hyde et al. 2020a).

Beltraniaceae mainly includes saprobic species that colonize tropical and subtropical ecosystems, such as decomposing leaf litter, soil, and submerged woody debris (Goh and Hyde 1996; Sakayaroj et al. 2005; Castañeda 2006; Shirouzu et al. 2010; Crous et al. 2014; Rajeshkumar et al. 2016; Lin et al. 2017a, b; Lichtemberg et al. 2019; Hyde et al. 2020b; Yasanthika et al. 2020). While most members serve as key decomposers, rare pathogenic species have been reported. *Hemibeltrania urbanodendrii* and *Pseudobeltrania angamosensis* were associated with leaf spots, respectively, on *Urbanodendron verrucosum* (Lauraceae) and *Virola gardneri* (Myristicaceae) in Brazil (Fernandes et al. 2007). *Beltrania rhombica* has been identified as a pathogenic fungus causing leaf spot disease in *Tibouchina semidecandra* in China (Shi et al. 2012). *Pseudobeltrania cedrelae* was identified as the causative fungus of pseudobeltrania spot disease affecting *Cedrela fissilis* in Brazil (Milagres et al. 2018).

*Beltrania* was introduced to accommodate *Beltrania rhombica* Penz., which was isolated from leaves of *Citrus limon* in Italy (Penzig 1882). As of 1 May 2025, Index Fungorum (http://www.indexfungorum.org/) lists 30 epithet records for *Beltrania*. Most species in *Beltrania* are saprophytic, but some are known as plant pathogens, for example, *Beltrania pseudorhombica* was reported as the causal agent of pistachio leaf and fruit spot in Arizona, United States (Lichtemberg et al. 2019). The genus is distinguished by mostly unbranched setae with radially lobed basal cells, unbranched conidiophores arising from basal cells of setae or from radially lobed cells, and biconic, spicate, or apiculate conidia with a hyaline transverse band (Penzig 1882; Zhang et al. 2022). The ethyl acetate extract of the freshwater fungus *Beltrania rhombica* demonstrated antibacterial activity against *Staphylococcus aureus* (MIC 0.98 mg/mL) and antifungal activity against *Candida albicans* (MIC 15.6 mg/mL) in a study on bioactive fungal metabolites (Rukachaisirikul et al. 2005).

*Beltraniella* was established with the type species *Beltraniella odinae* Subram., found on *Odina wodier* by Subramanian (1952) in India. The majority of *Beltraniella* species have been described based on their asexual morphs. A *Beltraniella* asexual morph was obtained by Hodges and Barr (1971) from pure cultures of *Pseudomassaria carolinensis* M.E. Barr & Hodges. Based on phylogenetic evidence, Jaklitsch et al. (2016) proposed the new combination *Beltraniella carolinensis*, with *Pseudomassaria carolinensis* reduced to synonymy. As of 1 May 2025, Index Fungorum lists 37 epithet records for *Beltraniella*. This genus is characterized by setiform conidiophores and polyblastic, sympodial, denticulate conidiogenous cells and turbinate or biconic conidia with a truncate base, rostrate apex, and a hyaline transverse band at the equatorial zone (Subramanian 1952; Shirouzu et al. 2010).

In this study, samples collected from southern China were analyzed. Six species were identified and classified through phylogenetic analysis of ITS and LSU sequences. These species are described and discussed based on both morphological characteristics and molecular data, thereby enriching the known species diversity within this fungal family.

## ﻿Materials and methods

### ﻿Strain isolation and preservation

Plant specimens with diseased or decaying leaves were collected from three provinces (Guizhou, Hainan, and Yunnan) of China during 2023–2024. After collection, the samples were labeled chronologically with collection time, location, and host plant species, then photographed and documented. Pure colonies were isolated using tissue culture techniques (Zhang et al. 2024; Li et al. 2025). From each sample, 5–7 infected or decaying leaves were cut from the edges into 5 × 5 mm fragments. The samples were surface sterilized by sequential immersion in 75% ethanol (1 min), sterile water (30 s), and 5% sodium hypochlorite solution (1 min). The fragments were rinsed three times with sterile water (30 s each), blotted dry with sterile filter paper, and subsequently transferred to PDA plates for incubation at 25 °C in darkness for 3 days. The hyphae were picked from the periphery of the PDA colonies and inoculated onto new PDA plates. The isolated strains were preserved in 15% sterilized glycerol and stored at 4 °C for future detailed studies. Voucher specimens are preserved in the Herbarium Mycologicum Academiae Sinicae, Chinese Academy of Sciences, Beijing (**HMAS**), and the Herbarium of the Department of Plant Pathology, Shandong Agricultural University, Taian, China (**HSAUP**). Corresponding ex-type living cultures were preserved in two culture collections: the Shandong Agricultural University Culture Collection (**SAUCC**) and the China General Microbiological Culture Collection Center (**CGMCC**).

The abbreviations of the genus names used in this study are as follows: *B.* = *Beltrania* and *Be.* = *Beltraniella*. To facilitate the delimitation of *Beltrania* and *Beltraniella* from other genera based on defining morphological characteristics, we provide dichotomous keys for Beltraniaceae.

### ﻿Morphological characterization

All isolates were inoculated on potato dextrose agar (PDA) medium, prepared by boiling 200 g peeled potatoes in 1 L distilled water for 30 min, then supplemented with 20 g dextrose and 20 g agar, and adjusted the pH to 7. The colony morphology, pigmentation, and growth rates of the isolates were recorded. PDA plate surfaces (obverse and reverse) were imaged after 14 days using a Canon Powershot G7X digital camera (Canon, Tokyo, Japan). Pine needles on water agar (WA) medium were used to describe morphological features such as the shape, size, and septation of the conidia. A stereomicroscope (Olympus SZ61, Olympus Corporation, Tokyo, Japan) and a microscope (Olympus BX53, Olympus Corporation, Tokyo, Japan) with differential interference contrast (DIC) were used to observe microstructures. Microscopic fungal structures were captured using BioHD-A20c color digital cameras (FluoCa Scientific, Shanghai, China) mounted on both stereomicroscopes and microscopes. Microstructures were measured randomly using Digimizer software v5.6.0 (https://www.digimizer.com), and their average size (av.) was calculated. The “n” represents the number of measurements.

### ﻿DNA extraction, amplification, and sequencing

Total genomic DNA was extracted from fresh fungal mycelia grown on potato dextrose agar (PDA) after 7 days using cetyltrimethylammonium bromide (CTAB) (Wang et al. 2023). Subsequently, the extracted DNA underwent polymerase chain reaction (PCR) targeting five segments, namely the internal transcribed spacer (ITS), the large subunit of ribosomal DNA (LSU), the partial translation elongation factor 1-alpha gene (*TEF1*-*α*), the RNA polymerase second largest subunit (*RPB2*), and the partial beta-tubulin gene (*TUB2*). For PCR amplification, five corresponding primer pairs were utilized: ITS5/ITS4, LR0R/LR5, EF1-728F/EF-2, 5f2/7cr, and Bt-2a/Bt-2b (Table 1). Although the GenBank submissions lack protein-coding genes, preventing their use in phylogenetic tree construction and comparative analysis with known species, we have provided the protein-coding sequences to facilitate future research. PCR amplification and purification were carried out in a 25 μL total volume containing 12.5 μL of 2 × Hieff Canace Plus PCR Master Mix (Cat. No. 10154ES03, Yeasen Biotechnology, Shanghai, China), 1 μL each of forward and reverse primers (TsingKe, Qingdao, China), 1 μL of template genomic DNA, and double distilled water to adjust the final volume. Amplified products were electrophoresed on a 1% agarose gel stained with Safe Red (Cat. Nos. RM02852 and RM19009, ABclonal Biotechnology Co., Ltd., Wuhan, China) and visualized under UV light (Ai et al. 2025). Target bands were excised and purified using a gel extraction kit (Cat. No. AE0101-C, Shandong Sparkjade Biotechnology Co., Ltd., Jinan, China) (Zhang et al. 2025). Purified PCR products were sequenced by Youkang Company Limited (Zhejiang, China). Sequence data were analyzed using MEGA v.7.0 (Kumar et al. 2016). The nucleotide sequences were submitted to the NCBI’s GenBank nucleotide database (https://www.ncbi.nlm.nih.gov/), and their accession numbers were listed in Table 2.

**Table 1.** Molecular markers and their PCR primers and programs used in this study.

Loci	PCR primers	Sequences (5′→3′)	PCR cycles	References
ITS	ITS5	GGA AGT AAA AGT CGT AAC AAG G	95 °C 5 min; (95 °C 30 s, 55 °C 30 s, 72 °C 1 min) × 35 cycles	White et al. 1990
	ITS4	TCC TCC GCT TAT TGA TAT GC		
LSU	LR0R	GTA CCC GCT GAA CTT AAG C	95 °C 5 min; (95 °C 30 s, 52 °C 30 s, 72 °C 1 min) × 35 cycles	Vilgalys and Hester 1990; Rehner and Samuels 1994
	LR5	TCC TGA GGG AAA CTT CG		
*TEF1*-*α*	EF1-728F	CAT CGA GAA GTT CGA GAA GG	95 °C 5 min; (95 °C 30 s, 55 °C 60 s, 72 °C 1 min) × 30 cycles	O’Donnell et al. 1998; Carbone and Kohn 1999
	EF2	GGA RGT ACC AGT SAT CAT GTT		
*RPB2*	5f2	GGG GWG AYC AGA AGA AGG C	95 °C 5 min; (94 °C 45 s, 55 °C 45 s, 72 °C 15 s) × 35 cycles	Liu et al. 1999
	7cr	CCC ATR GCT TGY TTR CCC AT		
*TUB2*	Bt-2a	GGT AAC CAA ATC GGT GCT GCT TTC	95 °C 5 min; (95 °C 30 s, 53 °C 30 s, 72 °C 1min) × 35 cycles	Glass and Donaldson 1995
	Bt-2b	ACC CTC AGT GTA GTG ACC CTT GGC		

**Table 2.** GenBank accession numbers of the taxa used in phylogenetic reconstruction.

Species	Culture accession	Host/substrate	Origin	GenBank accession numbers		References
				ITS	LSU	
*Beltrania aquatica*	MFLUCC 18-1418*	Submerged wood	Thailand	MZ351440	MZ351436	Phukhamsakda et al. 2022
*Beltrania dushanensis*	GZCC 18-0020 = DS 1-5*	Decaying seeds	China	MN252875	MN252882	Hyde et al. 2020a
*Beltrania krabiensis*	MFLUCC 16-0257*	Pandanaceae	Thailand	MH275048	MH260280	Tibpromma et al. 2018
*Beltrania liliiferae*	CGMCC 3.23464 = LC0063*	dead leaves of *Magnolia liliifera*	Thailand	OP022176	OP022172	Liu et al. 2024
*Beltrania liliiferae*	LC15869	dead leaves of *Magnolia liliifera*	Thailand	OP022177	OP022173	Liu et al. 2024
*Beltrania pseudorhombica*	CPC 23656 = CBS 138003 = CBS H-21717 *	*Pinus tabulaeformis*	China	KJ869158	KJ869215	Crous et al. 2014
*Beltrania pseudorhombica*	JSP 01-10 A 1.2	*Atta capiguara*	Brazil	KR093912	NA	Pereira et al. 2016
*Beltrania querna*	CBS 126097	NA	Spain	MH864016	MH875474	Vu et al. 2019
*Beltrania querna*	CBS 122.51	*Quercus ilex*, decayed leaf, in fountain	Italy	MH856775	MH868293	Vu et al. 2019
*Beltrania querna*	ICMP:15825	*Quercus ilex* dead leaf	New Zealand	EF029240	NA	Rajeshkumar et al. 2016
*Beltrania querna*	BCRC 34620	NA	China	GU905994	NA	Rajeshkumar et al. 2016
*Beltrania rhombica*	CBS 123.58 = IMI 0724323*	Sand near mangrove swamp	Mozambique	MH857718	MH869260	Liu et al. 2019
*Beltrania rhombica*	MFLUCC 15-0835 = MFLU 17-1261 = KNP 4-5	Decaying leaf	Thailand	MF580245	MF580252	Lin et al. 2017a
*Beltrania rhombica*	CPC 27482 = CBS 141507	*Acacia crassipes*	Malaysia	KX519515	KX519521	Rajeshkumar et al. 2016
*Beltrania rhombica*	CBS 121.50	*Eugenia aromatica*, seedling	Indonesia	NA	MH868082	Vu et al. 2019
*Beltrania rhombica*	10353	*Quercus myrsinaefolia* fallen leaves	Japan	NA	AB496423	NA
*Beltrania shenzhenica*	SAUCC 0061*	dead leaves of a broadleaf tree	China	MW784619	MW784621	Zhang et al. 2022
*Beltrania shenzhenica*	SAUCC 0065	dead leaves of a broadleaf tree	China	MW784620	MW784622	Zhang et al. 2022
*Beltrania sinensis*	YMF 1.05739*	*Quercus cocciferoides*	China	MN077365	MN077265	Zheng et al. 2020
*Beltrania sinensis*	JS43	*Quercus cocciferoides*	China	MN077366	MN077266	Zheng et al. 2020
*Beltrania sinensis*	JS101	*Quercus cocciferoides*	China	MN077363	MN077263	Zheng et al. 2020
*Beltrania sinensis*	JS260	*Fraxinus malacophylla*	China	MN077364	MN077264	Zheng et al. 2020
***Beltrania sinensis***	**SAUCC 3860**	***Litchi chinensis***	**China**	**PV577716**	**PV570360**	**This study**
***Beltrania sinensis***	**SAUCC 5483-1**	**Diseased leaves of *Quercus* sp.**	**China**	**PQ351195**	**PQ351411**	**This study**
***Beltrania veri***	CGMCC 3.28828 = SAUCC 8938-5A*	***Cinnamomum verum***	**China**	**PV577718**	**PV570362**	**This study**
***Beltrania veri***	**SAUCC 8938-5B**	***Cinnamomum verum***	**China**	**PV577719**	**PV570363**	**This study**
*Beltraniella acaciae*	CPC 29498 = CBS 142064*	*Acacia koa*	Hawaii	KY173389	KY173483	Crous et al. 2016
*Beltraniella botryospora*	TMQa1A18 = TUFC 10083*	*Quercus acuta*	Japan	NA	AB496426	Shirouzu et al. 2010
*Beltraniella brevis*	MFLU 19-2254 = DS 2-21 = GZCC 18-0081*	Decaying seeds	China	MN252876	MN252883	Hyde et al. 2020a
*Beltraniella brevis*	MFLU 19-2253 = DS 2-23 = GZCC 18-0082	Decaying seeds	China	MN252877	MN252884	Hyde et al. 2020a
*Beltraniella carolinensis*	Voucher 9502 (IFO)	*Persea borbonia* leaf	America	NA	DQ810233	Rajeshkumar et al. 2016
***Beltraniella danzhouensis***	CGMCC 3.28827 = SAUCC 6679-1*	**Decaying leaf**	**China**	**PQ351221**	**PQ351437**	**This study**
***Beltraniella danzhouensis***	**SAUCC 6834-2**	**Decaying leaf**	**China**	**PV577717**	**PV570361**	**This study**
*Beltraniella dujiangyanensis*	SAUCC 427003*	Decaying leaf	China	PP301351	PP301362	Liu et al. 2025
*Beltraniella dujiangyanensis*	SAUCC 427004	Decaying leaf	China	PP301352	PP301363	Liu et al. 2025
*Beltraniella endiandrae*	CPC 22193 = CBS 137976*	*Endiandra introrsa*	Australia	KJ869128	KJ869185	Crous et al. 2014
*Beltraniella fertilis*	MFLUCC 17-2136 = MFLU 17-1262 = MRC 2-1	Decaying leaf	Thailand	MF580246	MF580253	Lin et al. 2017b
*Beltraniella fertilis*	MFLUCC 17-2137 = MFLU 17-1263 = MRC 3BEL	Decaying leaf	Thailand	MF580247	MF580254	Lin et al. 2017b
*Beltraniella fertilis*	MFLUCC 17-2138 = MFLU 17-1264 = MRC 4-1	Decaying leaf	Thailand	MF580248	MF580255	Lin et al. 2017b
*Beltraniella fertilis*	MFLUCC 20-0119	Soil	Thailand	MT835158	MT835156	Yasanthika et al. 2020
*Beltraniella fertilis*	MFLUCC 19-0487 = MFLU 19-0982 = Seed 06	*Lithocarpus* sp.	Thailand	MT215489	MT215539	Perera et al. 2020
***Beltraniella fertilis***	**SAUCC 2153-2**	**Leaf litter**	**China**	**PQ351205**	**PQ351421**	**This study**
*Beltraniella hesseae*	BRIP 72433a*	*Digitaria ciliaris*	Australia	OP023124	OP023141	Tan and Shivas 2022
*Beltraniella humicola*	CBS 203.64*	soil	India	MH858416	MH870044	Vu et al. 2019
*Beltraniella jianfengensis*	SAUCC 639001*	Decaying leaf	China	PP301353	PP301364	Liu et al. 2025
*Beltraniella jianfengensis*	SAUCC 639002	Decaying leaf	China	PP301354	PP301365	Liu et al. 2025
***Beltraniella jianfengensis***	**SAUCC 2905-1A**	**Decaying leaf**	**China**	**PV577714**	**PV570358**	**This study**
***Beltraniella jianfengensis***	**SAUCC 2905-1B**	**Decaying leaf**	**China**	**PV577715**	**PV570359**	**This study**
*Beltraniella jiangxiensis*	CGMCC 3.23486 = LC3449*	*Camellia sinensis*	China	OP022178	OP022174	Liu et al. 2024
*Beltraniella jiangxiensis*	LC15868	*Camellia sinensis*	China	OP022179	OP022175	Liu et al. 2024
***Beltraniella jiangxiensis***	**SAUCC 3709-1**	**Decaying leaf**	**China**	**PQ351213**	**PQ351429**	**This study**
*Beltraniella myristicae*	SAUCC 638601*	Decaying leaf	China	PP301355	PP301366	Liu et al. 2025
*Beltraniella myristicae*	SAUCC 638602	Decaying leaf	China	PP301356	PP301367	Liu et al. 2025
*Beltraniella pandanicola*	MFLUCC 18-0121*	Pandanaceae	Thailand	MH275049	MH260281	Tibpromma et al. 2018
*Beltraniella podocarpi*	CPC 36783 = CBS 146633	*Podocarpus latifolius*	South Africa	MT373370	MT373353	Crous et al. 2020
*Beltraniella portoricensis*	CBS 856.70	*Persea borbonia*	NA	MH859981	MH871777	Vu et al. 2019
*Beltraniella portoricensis*	NFCCI 3993	*Mangifera indica*	India	KX519516	KX519522	Rajeshkumar et al. 2016
*Beltraniella portoricensis*	BCRC 34590	NA	China	GU905993	NA	Rajeshkumar et al. 2016
*Beltraniella pseudoportoricensis*	CPC34929 = CBS 145547*	*Podocarpus falcatus*	South Africa	MK876377	MK876416	Crous et al. 2019a
*Beltraniella ramosiphora*	MFLUCC 17-2582 = MFLU 17-2469 = LCG 10-2*	Decaying leaf	Thailand	MG717500	MG717502	Hyde et al. 2020b
*Beltraniella thailandica*	MFLUCC 16-0377 = KUMCC 17-0300*	Pandanaceae	Thailand	MH275050	MH260282	Tibpromma et al. 2018
*Beltraniella xinglongensis*	SAUCC 737701*	Decaying leaf	China	PQ325612	PQ325618	Liu et al. 2025
*Beltraniella xinglongensis*	SAUCC 737702	Decaying leaf	China	PQ325613	PQ325619	Liu et al. 2025
*Beltraniopsis longiconidiophora*	MFLUCC 17-2139 = MFLU 17-1265 = MRC 6-1*	Decaying leaf	Thailand	MF580249	MF580256	Lin et al. 2017b
*Beltraniopsis longiconidiophora*	MFLUCC 17-2140 = MFLU 17-1266 = MRC 12-2	Decaying leaf	Thailand	MF580250	MF580257	Lin et al. 2017b
*Beltraniopsis neolitseae*	CPC 22168 = CBS 137974*	*Neolitsea australiensis*	Australia	KJ869126	KJ869183	Crous et al. 2014
*Castanediella couratarii*	CBS 579.71*	Wood	Brazil	MH860269	MH872031	Hernández-Restrepo et al. 2016
*Hemibeltrania cinnamomi*	NFCCI 3695	*Cinnamomum malabathrum*	India	KT119564	KT119565	Rajeshkumar et al. 2016
*Hemibeltrania cinnamomi*	MFLUCC 17-2141 = MFLU 17-1267 = MRC 12-4	Decaying leaf	Thailand	MF580251	MF580258	Lin et al. 2017b
*Hemibeltrania cinnamomi*	NFCCI 3997	*Cinnamomum malabathrum*	India	KX519517	KX519523	Rajeshkumar et al. 2016
*Parapleurotheciopsis caespitosa*	CBS 519.93 = PREM 51686*	Leaf litter of *Syzygium cordatum* (Myrtaceae)	South Africa	MH862437	MH874086	Crous et al. 2018
*Parapleurotheciopsis inaequiseptata*	MUCL 41089 = INIFAT C98/30-1	Rotten leaf	Brazil	EU040235	EU040235	Crous et al. 2007
*Porobeltraniella porosa*	NFCCI 3994	Leaf litter	India	KX519518	KX519524	Rajeshkumar et al. 2016
*Porobeltraniella porosa*	NFCCI 3995	Leaf litter	India	KX519519	KX519525	Rajeshkumar et al. 2016
*Porobeltraniella porosa*	NFCCI 3996	Leaf litter	India	KX519520	KX519526	Rajeshkumar et al. 2016
*Pseudobeltrania cedrelae*	PF9	*Cedrela fissilis*	Brazil	MG559552	MG559562	Milagres et al. 2018
*Pseudobeltrania cedrelae*	COAD 2098*	*Cedrela fissilis*	Brazil	MG559548	MG559558	Milagres et al. 2018
*Pseudobeltrania lauri*	CPC 33589 = CBS 146025*	*Laurus azorica*	Spain	MN562097	MN567605	Crous et al. 2019b
*Pseudobeltrania ocoteae*	CBS 140664 = CPC 26219*	*Ocotea obtusata*	France	KT950856	KT950870	Crous et al. 2015
*Pseudosubramaniomyces fusisaprophyticus*	CBS 418.95	Leaf litter	Cuba	EU040241	EU040241	Crous et al. 2007
*Subsessila turbinata*	MFLUCC 15-0831 = MFLU 15-3271*	Decaying leaf	Thailand	KX762288	KX762289	Lin et al. 2017b

**Notes**: Species established in this study are shown in bold. Those marked “*” in the table are represented as ex-type or ex-epitype strains. NA: not available.

### ﻿Phylogenetic analyses

**Figure 1.**
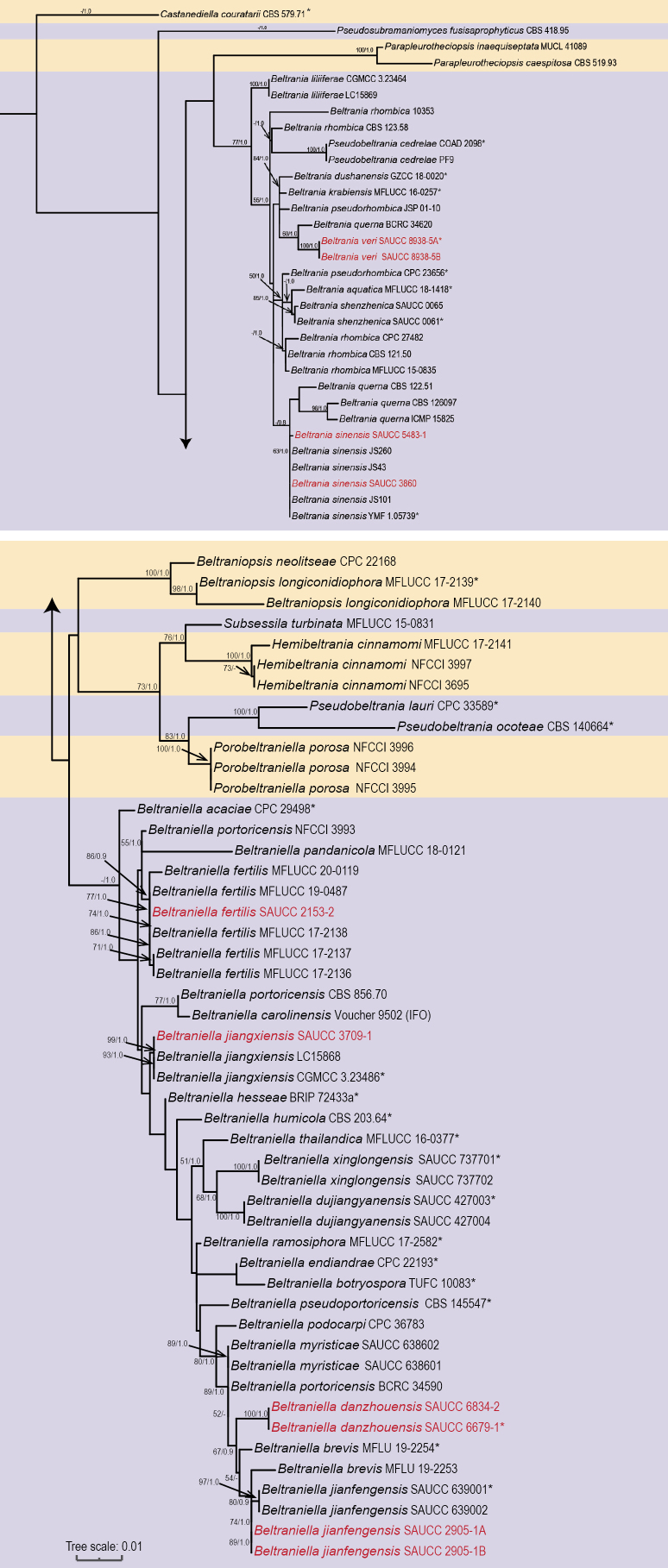
Phylogenetic tree generated from maximum likelihood analysis based on combined ITS and LSU sequence data of Beltraniaceae. Bootstrap support values are shown as ML ≥ 50% first, followed by BI ≥ 0.80. *Castanediella couratarii* (CBS 579.71) was used as the outgroup taxon. Those marked “*” in the tree represent ex-type or ex-epitype strains. Strains isolated in this study are indicated in red.

All sequences were initially aligned using the MAFFT v.7 online service (http://mafft.cbrc.jp/alignment/server/) and then manually corrected in MEGA v7.0 (Katoh et al. 2019). GenBank submissions lack protein-coding genes. The concatenated ITS and LSU sequences were used for maximum likelihood (ML; executed in RAxML-HPC2 on XSEDE v.8.2.12) and Bayesian inference (BI; executed in MrBayes v.3.2.7a with 16 threads on Linux) (Zhang et al. 2024). For ML analyses, 1000 rapid bootstrap replicates and the GTR+G+I model with default parameters were used. For BI analyses, we used a fast bootstrap algorithm with an automatic stop option and employed MrModeltest v.2.3 to determine the best evolutionary model for each partition (Nylander 2004; Zhang et al. 2024). The GTR+I+G model for both ITS and LSU was selected and incorporated into the analyses. Bayesian inference posterior probabilities (BIPP) were estimated through Markov Chain Monte Carlo (MCMC) sampling (Rannala and Yang 1996; Zhaxybayeva and Gogarten 2002). The sequence analysis was performed over 5,000,000 generations, yielding 5,842 trees. After discarding 1,460 trees during the burn-in phase, the remaining trees were used to calculate posterior probabilities in the consensus trees. The phylogenetic trees were visualized and optimized using the Interactive Tree of Life (ITOL) platform (https://itol.embl.de/), and the final layout was refined in Adobe Illustrator CS6. The isolates in this study are marked in red in the phylogenetic tree (Fig. 1). We also present separate phylogenetic trees for *Beltrania* and *Beltraniella* (Suppl. material 1), along with dichotomous keys for *Beltrania* (Suppl. material 2) and *Beltraniella* (Suppl. material 3) in the Suppl. material.

## ﻿Results

### ﻿Phylogenetic analyses

Based on the ITS sequence data, four strains were initially identified as belonging to *Beltrania* and six strains as *Beltraniella*. A combined phylogenetic analysis of ITS and LSU gene regions was then conducted using ML and BI approaches to determine the precise phylogenetic positions of these strains. The dataset comprised 81 sequences representing 42 species, with *Castanediella couratarii* (CBS 579.71) as the outgroup. The final alignment consisted of 1384 characters from the combined ITS and LSU sequences, including gaps, spanning positions 1–589 (ITS) and 590–1384 (LSU). The ML optimization likelihood was calculated as −5572.183054. In the sequence alignment, 1384 distinct patterns were identified, with 10.35% of positions undetermined or containing gaps. In BI analysis, the alignment contained 397 unique site patterns (ITS: 242; LSU: 155). Since the ML and BI trees showed identical topologies, only the ML tree is presented (Fig. 1). By combining morphological characteristics and molecular phylogenetic analyses, the ten strains in this study were identified.

### ﻿Taxonomy

#### ﻿ Beltrania sinensis

Taxon classificationFungiAmphisphaerialesBeltraniaceae

Hua Zheng, X.Q. Yang, J.S. Deng, J.P. Xu & Z.F. Yu, Int. J. Syst. Evol. Microbiol. 70(2): 1180 (2020)

351B4765-AFEE-5722-A155-2FD7FD0EA5E3

Fig. 2

##### Description.

***Sexual morph***: Undetermined. ***Asexual morph***: Setae numerous, erect, arising from radially lobed basal cells, straight or slightly flexuous, unbranched, thick-walled, verrucose, medium to dark brown, paler at apex, 135–242 µm long, 4.2–5.7 µm wide, tapering to a pointed apex, or arising from a dark brown, swollen, radially lobed basal cell, 11.9–17.6 μm in diameter. Imperfect setae single, flexuous, septate, verrucose, pale brown, swollen at the base. Conidiophores macronematous, mononematous, erect, subcylindrical or clavate, unbranched, pale brown, smooth, straight to flexuous, 64–134 × 4.4–6.7 µm. Conidiogenous cells polyblastic, integrated, terminal, sympodial, with flat-tipped denticles, subhyaline to pale brown, smooth, 9.0–14.3 µm (av. = 12.3 μm, n = 10) long, 4.0–5.2 µm (av. = 4.7 μm, n = 10) wide. Separating cells subhyaline to pale brown, finely roughened, aseptate, oval or obovoid, 9.2–13.2 µm (av. = 11.1 µm, n = 22) long, 4.4–7.0 µm (av. = 5.7 µm, n = 22) wide in the broadest part, 1-denticulate at each end. Conidia arise directly from conidiogenous cells or from separating cells, acrogenous, biconic, aseptate, smooth, pale brown with a hyaline to subhyaline equatorial transverse band, with distinct granules, rounded or 1-denticulate at base, 29.3–34.4 µm (av. = 32.1 µm, n = 21) long including appendage, 7.2–10.3 µm (av. = 8.3 µm, n = 21) wide in the broadest part, apical appendages 7.2–10.1 µm (av. = 9.1 µm, n = 21) long, tapering to an acutely rounded tip.

**Figure 2.**
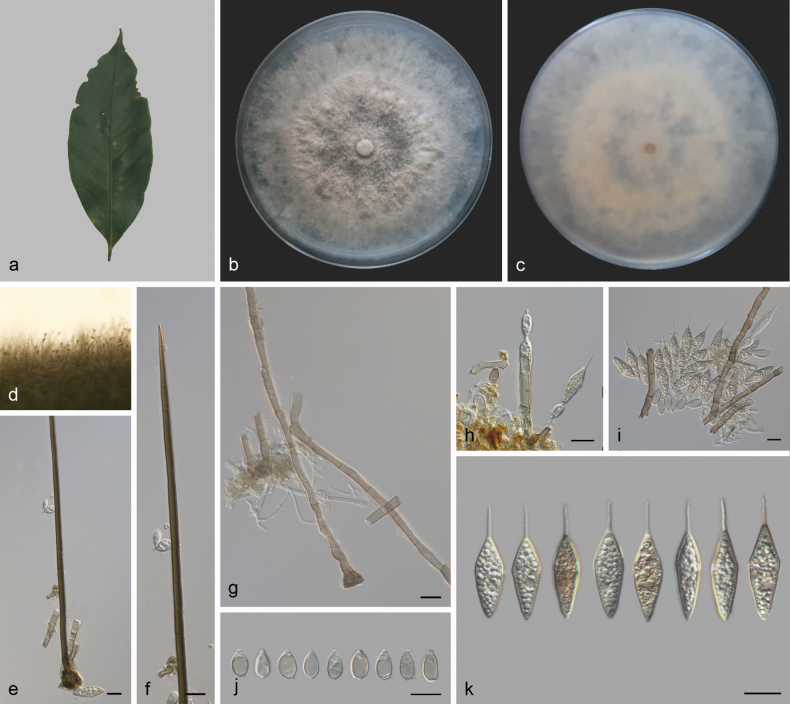
*Beltrania sinensis* (SAUCC 3860). a. Leaf of host plant; b, c. Upper and reverse views of cultures on PDA after 14 days of inoculation at 25 °C; d. Conidiophores and setae on a pine needle on WA; e. Basal part of seta; f. Apex of seta; g. Imperfect setae; h. Conidiophores, conidiogenous cells, separating cell and conidia; i. Separating cells and conidia; j. Separating cells; k. Conidia. Scale bars: 10 μm (e–k).

##### Culture characteristics.

Colonies on PDA at 25 °C in darkness, occupying an entire 90 mm Petri dish in 14 d. Surface pale brown and white, prominent in the middle and flat at the edges. Reverse pale yellow in the middle, with the edges being medium white.

##### Materials examined.

China • Hainan Province, Ledong Li Autonomous County, Jianfengling National Forest Park, on leaves of *Litchi chinensis*, 12 April 2023, Q.Y. Liu, (HSAUP 3860, new host and locality re­cord), living cultures SAUCC 3860; China • Guizhou Province, Qiannan Buyei and Miao Autonomous Prefecture, Longli County, Longshan Town, Longxi Ave­nue, on diseased leaves of *Quercus* sp., Q.Y. Liu, (HSAUP 5483-1, new locality record), living cultures SAUCC 5483-1.

##### GeneBank numbers.

SAUCC 3860: ITS = PV577716, LSU = PV570360, *RPB2* = PV420882, *TEF1*-*α* = PV420893, *TUB2* = PV420901; SAUCC 5483-1: ITS = PQ351195, LSU = PQ351411, *RPB2* = PV420881, *TEF1*-*α* = PV420894, *TUB2* = PV420902.

##### Notes.

*Beltrania sinensis* was originally described from the root of *Quercus cocciferoides* and *Fraxinus malacophylla* in Jianshui County, Yunnan Province, China (Zheng et al. 2020). In the present study, two strains (SAUCC 3860 and SAUCC 5483-1) are clustered with the *B. sinensis* (YMF 1.05739, JS101, JS260, and JS43) clade in the combined phylogenetic tree (Fig. 1). Our strains (SAUCC 3860 and SAUCC 5483-1) were similar to *B. sinensis* (YMF 1.05739) in ITS (with 100% and 99.81% sequence identity, respectively) and LSU (with 100% and 99.87% sequence identity, respectively). Morphologically, our two strains were similar to *B. sinensis* (YMF 1.05739) by setae and separating cells (135–242 × 4.2–5.7 vs. 155–269 × 4–5 μm; 9.2–13.2 × 4.4–7.0 vs. 8.5–13.0 × 5.2–6.0 μm). Thus, we consider the isolated strains as *B. sinensis*. Additionally, *B. sinensis* (YMF 1.05739, JS101, JS260, and JS43) were found in the roots in Yunnan Province, China. Our two strains represent new records: SAUCC 3860 was first discovered on *Litchi chinensis* in Hainan Province (new host and locality record), while SAUCC 5483-1 was first recorded in Guizhou Province (new locality record) (Zheng et al. 2020).

#### ﻿ Beltrania veri

Taxon classificationFungiAmphisphaerialesBeltraniaceae

D.H. Li, J.W. Xia & X.G. Zhang sp. nov.

AD160D2A-A3B1-55C8-8A83-8B5A744582BD

857568

Fig. 3

##### Etymology.

Named after the species epithet of the host plant *Cinnamomum verum*.

##### Holotype.

HMAS 354000.

##### Description.

***Sexual morph***: Undetermined. ***Asexual morph***: Setae numerous, arising from radially lobed basal cells, straight or flexuous, unbranched, single, thick-walled, coarsely verrucose, pale brown to dark brown, tapering to a pointed apex, or arising from a dark brown, swollen, radially lobed basal cell, 193–390.5 μm long, 12.0–20.8 μm in diameter. Imperfect setae single, slightly flexuous, septate, verrucose, pale brown, swollen at the base. Conidiophores macronematous, simple, thin-walled, subhyaline to pale yellow, arising from basal cells of setae, 112–182 × 4.7–5.0 µm. Conidiogenous cells polyblastic, holoblastic, integrated, determinate, terminal, geniculate, cylindrical, hyaline to subhyaline, smooth, 8.2–26.2 × 4.0–6.4 μm. Conidia arise directly from conidiogenous cells, acrogenous, biconic, aseptate, smooth, pale yellow, with distinct granules, rounded or 1-denticulate at base, 15.5–21.5 µm (av. 18.2 ± 1.8 µm, n = 26) long including appendage, 8.0–10.7 µm (av. 9.3 ± 0.8 µm, n = 21) wide in the broadest part, apical appendages 1.4–2.3 µm (av. 1.8 µm, n = 19) long.

**Figure 3.**
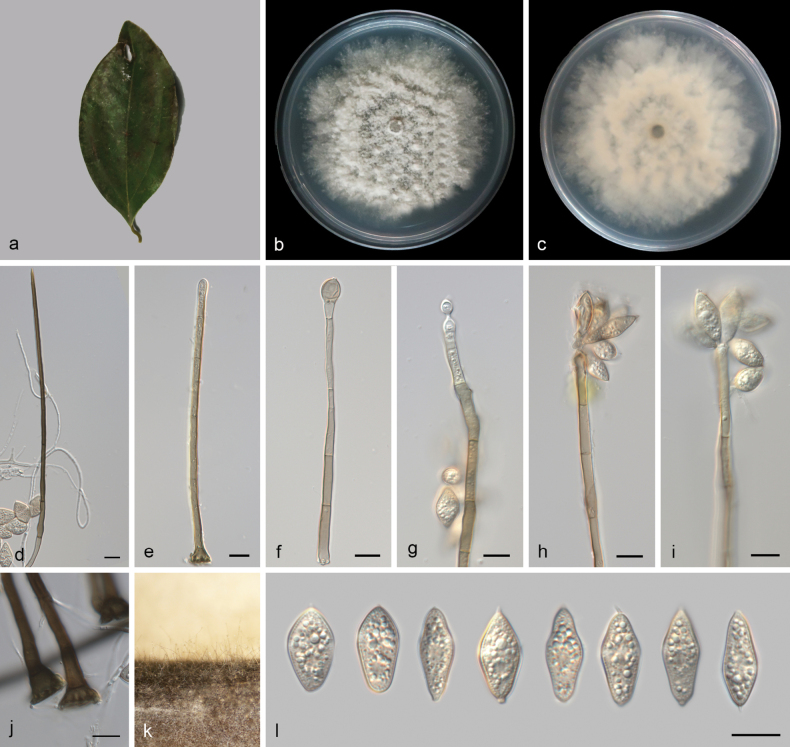
*Beltrania veri* (SAUCC 8938-5A). a. Leaf of host plant; b, c. Upper and reverse views of cultures on PDA after 14 days of inoculation at 25 °C; d. Seta and conidia; e. Imperfect seta; f–i. Conidiophores, conidiogenous cells, and conidia; j. Basal part of setae; k. Conidiophores and setae on a pine needle in WA; l. Conidia. Scale bars: 10 μm (d–j, l).

##### Culture characteristics.

Colonies on PDA at 25 °C in darkness, reaching 71 mm in diameter in 14 d. Surface white, dense, and raised in the middle. Reverse pale yellow.

##### Material examined.

China • Yunan Province, Puer City, Simao District, Puer Sun River Forest Park, on leaves of *Cinnamomum verum*, 15 May 2023, Q.Y. Liu, (HMAS 354000, holotype), ex-type living culture CGMCC 3.28828 = SAUCC 8938-5A; Ibid., (HSAUP 8938-5B, paratype), living culture SAUCC 8938-5B.

##### GeneBank numbers.

SAUCC 8938-5A: ITS = PV577718, LSU = PV570362, *RPB2* = PV420883, *TEF1*-*α* = PV420897, *TUB2* = PV420905; SAUCC 8938-5B: ITS = PV577719, LSU = PV570363, *RPB2* = PV420884, *TEF1*-*α* = PV420898, *TUB2* = PV420906.

##### Notes.

Phylogenetic analysis showed that two isolates (SAUCC 8938-5A and SAUCC 8938-5B) of *Beltrania veri* were closely related to *B. querna* (BCRC 34620). *B. querna* (BCRC 34620) only has ITS sequence data (Rajeshkumar et al. 2016). Phylogenetically, *B. veri* (SAUCC 8938-5A, ex-type strain) is closely related to *B. querna* (BCRC 34620) and shows 7/493 differences in ITS. Morphologically, our specimens differ from *B. querna* by their shorter apical appendages (1.4–2.3 µm vs. 4–9 µm), longer setae (193–390.5 μm vs. 125–185 μm), and shorter conidia (15.5–21.5 μm vs. 23–28 μm) (Barbosa et al. 2009). Thus, based on the guidelines of Chethana et al. (2021), we describe this fungus as a new species.

#### ﻿ Beltraniella danzhouensis

Taxon classificationFungiAmphisphaerialesBeltraniaceae

D.H. Li, J.W. Xia & X.G. Zhang sp. nov.

E39BEF4F-FB1E-5D76-A32D-8F030E85F550

857852

Fig. 4

##### Etymology.

The epithet “danzhouensis” denotes the geographical origin of the strains, namely Danzhou City.

##### Holotype.

HMAS 354001.

##### Description.

***Sexual morph***: Undetermined. ***Asexual morph***: Setae numerous, erect, arising from radially lobed basal cells, straight or flexuous, unbranched, single or in small groups, thick-walled, smooth, pale brown, paler towards apex, 140–208 μm long, 4–7.4 μm wide, tapering to a pointed apex, or arising from a pale brown, swollen, radially lobed basal cell, 12–25 μm in diameter. Conidiophores macronematous, simple or branched, septate, smooth-walled, swollen at the base, subhyaline to pale brown, thin-walled, arising from basal cells of setae or from separate, 22–47 μm long, 3–7 μm wide. Conidiogenous cells holoblastic, integrated, determinate, terminal, cylindrical, oblong, hyaline to subhyaline, smooth, 11–21 × 5.7–6.0 μm (av. = 15.91 ± 4.31 × 5.30 ± 0.53 μm). Separating cells clavate, thin-walled, smooth, hyaline, 1-denticulate at each end, 12.18–13.59 × 3.7–3.9 μm (av. = 12.90 ± 0.99 × 3.84 ± 0.01 μm). Conidia arise directly from conidiogenous cells or from separating cells, aggregated, acrogenous, simple, dry, straight, thin-walled, turbinate to obpyriform, rostrate to pointed at proximal end, rounded at distal end, hyaline to subhyaline, aseptate, 17.6–22.6 × 5.1–7.8 μm (av. = 20.48 ± 1.26 × 5.96 ± 0.71 μm).

**Figure 4.**
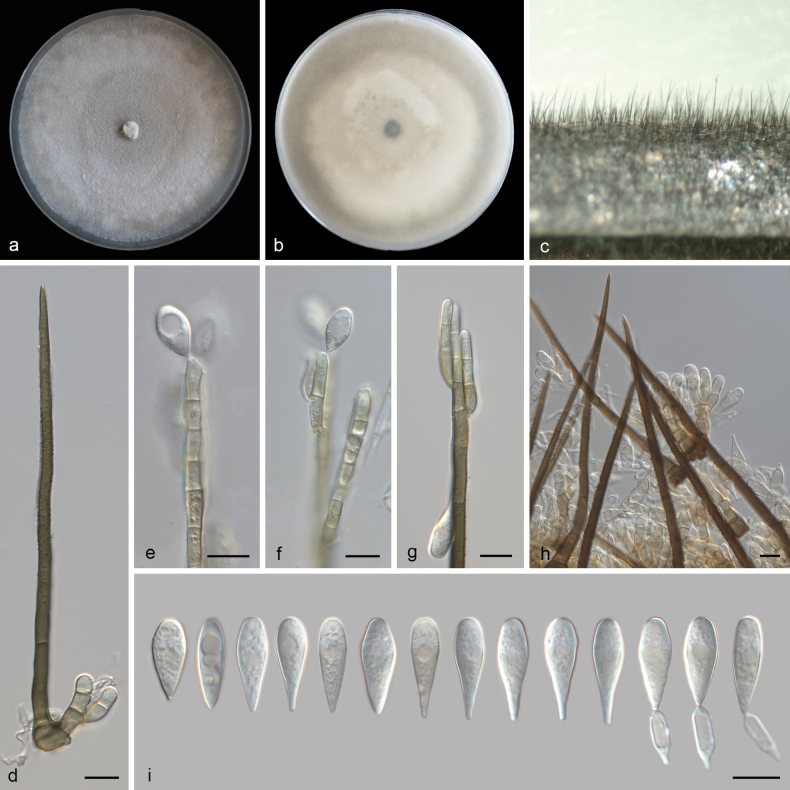
*Beltraniella danzhouensis* (SAUCC 6679-1). a, b. Upper and reverse views of cultures on PDA after 14 days of inoculation at 25 °C; c. Conidiophores and setae on a pine needle in WA; d. Seta and conidiophore; e–g. Conidiophores, conidiogenous cells, and conidia; h. Setae, conidiophores, conidiogenous cells, and conidia; i. Conidia and conidia with separating cells. Scale bars: 10 μm (d–i).

##### Culture characteristics.

Colonies on PDA at 25 °C in darkness, occupying an entire 90 mm Petri dish in 14 d. Surface greyish white, granular, dense. Reverse cream-colored in the center and pale brown margin.

##### Material examined.

China • Hainan Province, Danzhou City, Hainan Tropical Botanical Garden, on decaying leaves, 10 April 2023, Q.Y. Liu, (HMAS 354001, holotype), ex-type living culture CGMCC 3.28827 = SAUCC 6679-1; Ibid., (HSAUP 6834-2, paratype), living culture SAUCC 6834-2.

##### GeneBank numbers.

SAUCC 6679-1: ITS = PQ351221, LSU = PQ351437, *RPB2* = PV420888, *TEF1*-*α* = PV420895, *TUB2* = PV420903; SAUCC 6834-2: ITS = PV577717, LSU = PV570361, *RPB2* = PV420889, *TEF1*-*α* = PV420896, *TUB2* = PV420904.

##### Notes.

Based on the phylogenetic analyses using sequences of two genes (ITS and LSU), *Beltraniella danzhouensis* belongs to the large clade, where it shows a relationship with *Be. brevis* and *Be. jianfengensis*. *Be. danzhouensis* differs from *Be. brevis* (MFLU 19-2254, ex-type strain) by its production of shorter separating cells (12.18–13.59 × 3.7–3.9 µm vs. 11–18 × 3.4–4.1 µm) and distinct morphological features. Specifically, *Be. danzhouensis* (SAUCC 6679-1) produced thin-walled, turbinate to obpyriform conidia lacking a hyaline supraequatorial transverse band, with clavate separating cells, whereas *Be. brevis* (MFLU 19-2254) has conidia with a hyaline supraequatorial transverse band and fusiform separating cells, and the presence of 14 distinct nucleotide positions (11/548 in ITS, 3/766 in LSU) (Hyde et al. 2020a). *Be. danzhouensis* differs from *Be. jianfengensis* (SAUCC 639001, ex-type strain) by its production of shorter conidiophores (22–47 μm vs. 171.8–254.9 μm and 20.1–57.2 μm) and bigger conidiogenous cells (11–21 × 5.7–6.0 µm vs. 9.2–15.3 × 2.2–5.0 μm) and the presence of 14 distinct nucleotide positions (8/567 in ITS, 6/781 in LSU) (Liu et al. 2025). Consequently, *Be. danzhouensis* was classified as a new species within the genus *Beltraniella* through a combination of phylogenetic analysis and morphological comparisons.

#### ﻿ Beltraniella fertilis

Taxon classificationFungiAmphisphaerialesBeltraniaceae

Heredia, R.M. Arias, M. Reyes & R.F. Castañeda, Fungal Diversity 11: 100 (2002)

5481B643-1D73-5792-B12A-C22465A5ED1F

Fig. 5

##### Description.

***Sexual morph***: Undetermined. ***Asexual morph***: Setae numerous, erect, arising from radially lobed basal cells, straight or flexuous, unbranched, single, thick-walled, verrucose, dark brown, 130–189 μm long, 5.4–8.6 μm wide at the base, tapering to apointed apex, or arising from a dark brown, swollen, radially lobed cell, 12.9–22.8 μm in diameter. Conidiophores macronematous, sometimes setiform, single, straight, partly verrucose, smooth-walled, 21.9–60.7 μm long, 3.2–5.2 μm wide (av. = 39 μm × 4.2, n = 11), sometimes branched at the apical region, subhyaline to pale yellow, paler towards a pointed apex. Conidiogenous cells monoblastic to polyblastic, integrated, terminal, geniculate, cylindrical, hyaline to subhyaline, smooth, 10–12 × 2.4–5.2 μm (av. = 11.39 ± 1.05 × 6.00 ± 0.87 μm). Separating cells ovoid or fusiform, thin-walled, smooth, hyaline to subhyaline, 1-denticulate at each end, 8.9–11.9 µm (av. = 10.4 μm, n = 25) long, 3.2–4.6 µm (av. = 3.9 μm, n = 25) wide in the broadest part. Conidia acrogenous, simple, dry, straight, smooth, thin-walled, turbinate to obpyriform, rostrate to pointed at proximal end, rounded at distal end, hyaline to subhyaline, 17.8–23.7 µm long, 3.9–8.1 µm wide (av. = 20.28 × 6.25 μm, n = 25) in the broadest part.

**Figure 5.**
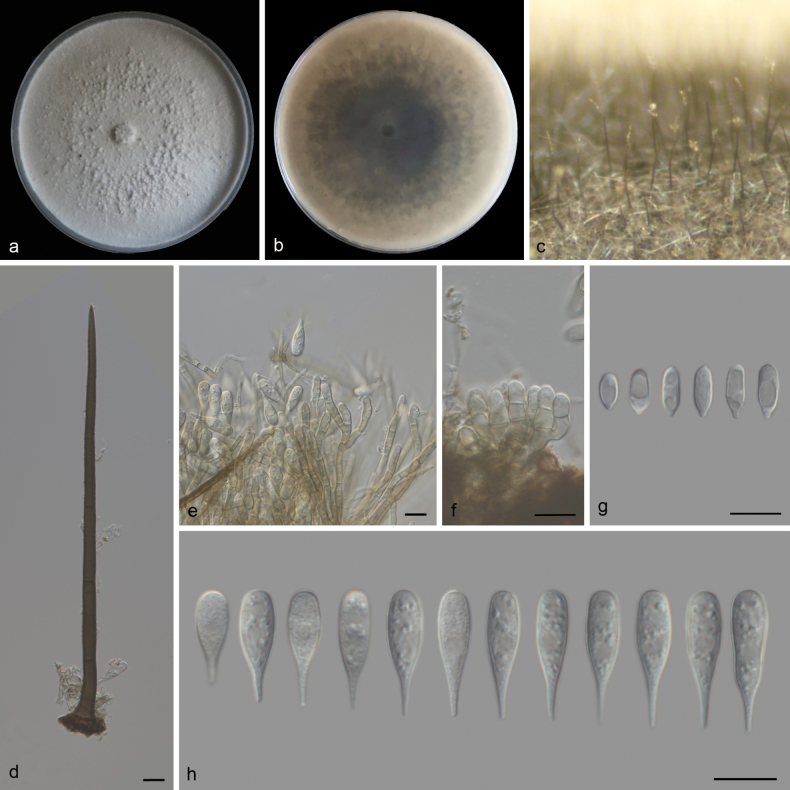
*Beltraniella fertilis* (SAUCC 2153-2). a, b. Upper and reverse views of cultures on PDA after 14 days of inoculation at 25 °C; c. Conidiophores and setae on a pine needle in WA; d. Seta; e. Conidiophores, conidiogenous cells, and conidia; f. Conidiophores and conidiogenous cells; g. Separating cells; h. Conidia. Scale bars: 10 μm (d–h).

##### Culture characteristics.

Colonies on PDA at 25 °C in darkness, occupying an entire 90 mm Petri dish in 14 d. Surface greyish white, prominent. Reverse tanned in the middle, with the edges being medium brown.

##### Materials examined.

China • Yunnan Province, Xishuangbanna Primitive For­est Park, on leaf litter, 17 March 2023, Q.Y. Liu, (HSAUP 2153-2, new locality re­cord), living cultures SAUCC 2153-2.

##### GeneBank numbers.

SAUCC 2153-2: ITS = PQ351205, LSU = PQ351421, *RPB2* = PV420886, *TEF1*-*α* = PV420890, *TUB2* = PV420899.

##### Notes.

Existing strains of *Beltraniella fertilis* (MFLUCC 17-2136, MFLUCC 17-2137, MFLUCC 17-2138, MFLUCC 19-0487, and MFLUCC 20-0119) and our isolate (SAUCC 2153-2) clustered with good support (ML = 86%, BI = 0.9) in the phylogenetic tree. A comparison of nucleotides shows the ITS and LSU loci of our isolate are identical to those of *Be. fertilis* (MFLUCC 17-2136, MFLUCC 17-2137, MFLUCC 17-2138, and MFLUCC 19-0487). Our isolate is similar to *Be. fertilis* in its setae, conidiophores, and conidia. However, the conidial size of MFLUCC 20-0119 is smaller than that of the others. No median transverse band of lighter pigment was observed in the conidia of our isolate and MFLUCC 20-0119. These morphological variations are likely caused by differences in environmental conditions (Francisco et al. 2019). Based on morphological characteristics and phylogenetic analysis, this study is the first report of *Be. fertilis* in China (Heredia et al. 2002; Lin et al. 2017b; Perera et al. 2020; Yasanthika et al. 2020).

#### ﻿ Beltraniella jianfengensis

Taxon classificationFungiAmphisphaerialesBeltraniaceae

W.W. Liu, C.Z. Yin, Z.X. Zhang & X.G. Zhang, MycoKeys 116: 132 (2025)

BFC51BC5-AA82-53EC-8F1B-1F9115AC39AE

Fig. 6

##### Description.

***Sexual morph***: Undetermined. ***Asexual morph***: Setae numerous, erect, straight or flexuous, unbranched, single or in small groups, thick-walled, verrucose, dark brown, 102–165 μm long, 3.9–9.3 μm wide at the base, tapering to a pointed apex, or arising from a dark brown, swollen, radially lobed basal cell, 8.9–15.6 μm in diameter. Imperfect setae single, flexuous, septate, verrucose, thick-walled, pale brown, up to 115 μm long, swollen at the base. Conidiophores macronematous, short, simple or branched, septate, verrucose, swollen at the base, subhyaline to pale brown, thin-walled, arising from basal cells of setae, 26–87 μm long, 5.9–9.7 μm wide. Conidiogenous cells polyblastic, integrated, determinate, terminal, cylindrical, oblong, hyaline to subhyaline, verrucose, 6.3–33.3 µm (av. = 16.9 μm, n = 16) long, 4.2–6.9 µm (av. = 5.8 μm, n = 16) wide at the base. Separating cells fusiform, thin-walled, smooth, hyaline to subhyaline, 1-denticulate at each end, 11–14 µm long, 3.1–4.7 µm wide in the broadest part. Conidia arise directly from conidiogenous cells or from separating cells, aggregated, acrogenous, simple, dry, straight, smooth, thin-walled, turbinate to obpyriform, rostrate to pointed at proximal end, rounded at distal end, hyaline, 10.82–32.82 µm (av. = 25.2 μm, n = 26) long, 4.1–8.3 µm (av. = 7.1 μm, n = 26) wide in the broadest part.

**Figure 6.**
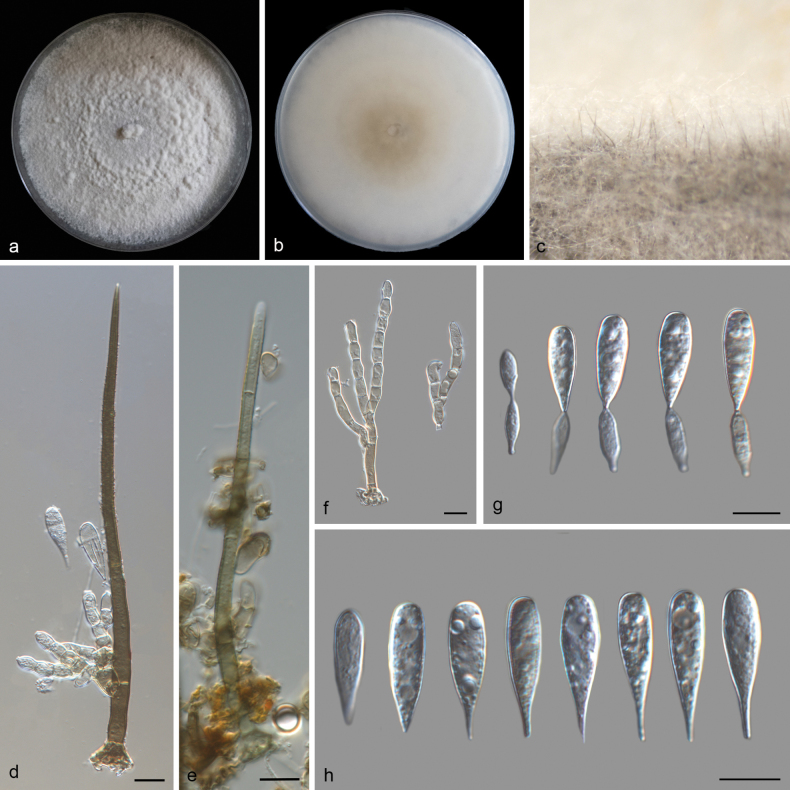
*Beltraniella jianfengensis* (SAUCC 2905-1A). a, b. Upper and reverse views of cultures on PDA after 14 days of inoculation at 25 °C; c. Conidiophores and setae on a pine needle in WA; d. Seta, conidiophores, conidiogenous cells, and conidia; e. Imperfect seta; f. Conidiophores and conidiogenous cells; g. Conidia with separating cells; h. Conidia. Scale bars: 10 μm (d–h).

##### Culture characteristics.

Colonies on PDA at 25 °C in darkness, occupying an entire 90 mm Petri dish in 14 d. Surface white, prominent. Reverse pale yellow in the middle with the edges being medium white.

##### Materials examined.

China • Hainan Province, Lingshui Li Autonomous County, Diaoluo Mountain National Forest Park, on decaying leaves, 9 April 2023, Q.Y. Liu, HSAUP 2905-1A, living cultures SAUCC 2905-1A; Ibid., HSAUP 2905-1B, living culture SAUCC 2905-1B.

##### GeneBank numbers.

SAUCC 2905-1A: ITS = PV577714, LSU = PV570358, *RPB2* = PV578285, *TEF1*-*α* = PV578289, *TUB2* = PV578287; SAUCC 2905-1B: ITS = PV577715, LSU = PV570359, *RPB2* = PV578286, *TEF1*-*α* = PV578290, *TUB2* = PV578288.

##### Notes.

In the phylogenetic analysis, our isolates (SAUCC 2905-1A and SAUCC 2905-1B) clustered with *Beltraniella brevis* (MFLU 19-2253) and *Be. jianfengensis* (SAUCC 639001 and SAUCC 639002) with high support (ML = 80%, BI = 0.9). Our isolates (SAUCC 2905-1A and SAUCC 2905-1B) and *Be. jianfengensis* (SAUCC 639001, ex-type strain) were similar in ITS (2/567) and LSU (1/781). Morphologically, our isolates (SAUCC 2905-1A and SAUCC 2905-1B) are similar to *Be. jianfengensis* (SAUCC 639001) by separating cells (SAUCC 2905-1A and SAUCC 2905-1B: 11–14 × 3.1–4.7 µm; SAUCC 639001: 10.7–14.7 × 2.8–5.5 µm) and conidia (SAUCC 2905-1A and SAUCC 2905-1B: 10.82–32.82 × 4.1–8.3 µm; SAUCC 639001: 17.1–23.6 × 3.6–9.5 µm), and conidia lacking a hyaline transverse band. *Be. jianfengensis* (SAUCC 639001 and SAUCC 639002) was originally discovered on decaying leaves in Jianfengling National Forest Park, Hainan Province, China (Liu et al. 2025). Based on morphological characteristics and phylogenetic analysis, we report our isolates (SAUCC 2905-1A and SAUCC 2905-1B) as *Be. jianfengensis* from decaying leaves in Diaoluo Mountain National Forest Park, Hainan Province, China.

#### ﻿ Beltraniella jiangxiensis

Taxon classificationFungiAmphisphaerialesBeltraniaceae

P. Razaghi, M. Raza & L. Cai, Fungal Diversity 124: 47 (2024)

339E9C78-3BEC-5A6A-B1A1-2A1594BEA1A0

Fig. 7

##### Description.

***Sexual morph***: Undetermined. ***Asexual morph***: Setae numerous, arising from radially lobed basal cells, straight or flexuous, unbranched, single, thick-walled, coarsely verrucose, tanned, 117–169 μm long, 5.14–5.46 μm wide, tapering to a pointed apex, or arising from a dark brown, swollen, radially lobed basal cell, 8.2–14.6 μm in diameter. Conidiophores macronematous, simple or branched at apical regions, thin-walled, swollen at the base, subhyaline to pale yellow, arising from basal cells of setae or from separate, 32–69 × 4–6 µm. Conidiogenous cells polyblastic, integrated, determinate, terminal, geniculate, denticulate, cylindrical, oblong, hyaline to subhyaline, smooth, 12–29 × 3.8–9.3 μm (av. = 19.74 ± 5.82 × 6.32 ± 1.95 μm). Conidia simple, dry, straight, thin-walled, turbinate to obpyriform, rostrate to pointed at proximal end, rounded at distal end, hyaline to subhyaline without a hyaline transverse band, aseptate, 22.3–26.1 × 5.1–8.2 μm (av. = 23.94 ± 1.09 × 6.23 ± 0.78 μm).

**Figure 7.**
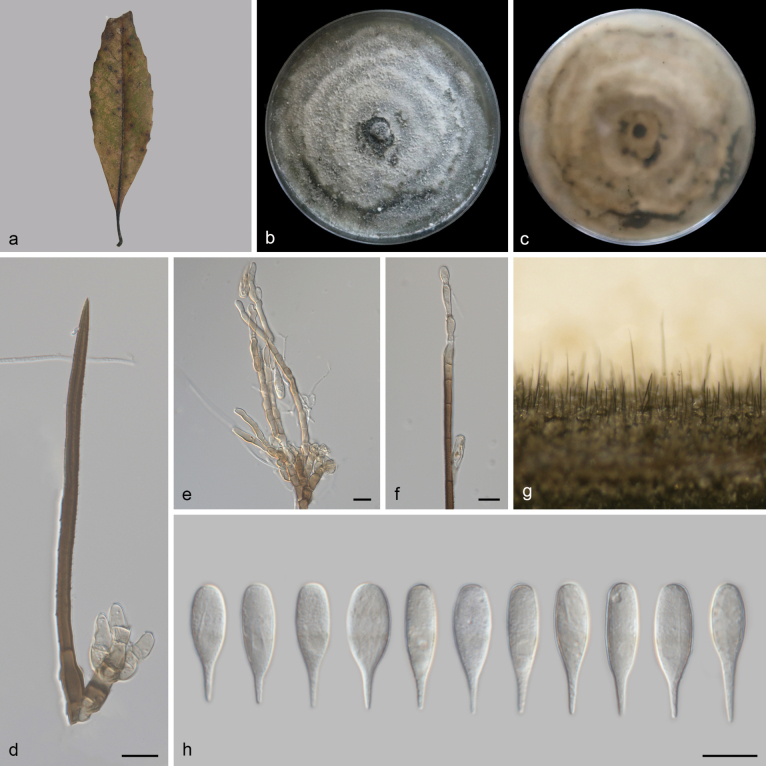
*Beltraniella jiangxiensis* (SAUCC 3709-1). a. Leaf of host plant; b, c. Upper and reverse views of cultures on PDA after 14 days of inoculation at 25 °C; d. Seta, conidiophore, and conidiogenous cells; e, f. Conidiophores and conidiogenous cells; g. Conidiophores and setae on a pine needle on WA; h. Conidia. Scale bars: 10 μm (d–f, h).

##### Culture characteristics.

Colonies on PDA at 25 °C in darkness, occupying an entire 90 mm Petri dish in 14 d. Surface greyish white, flat, dense, concentric rings. Reverse cream-colored or pale brown concentric rings.

##### Materials examined.

China • Hainan Province, Ledong Li Autonomous Coun­ty, Jianfeng Town, on decaying leaves, 11 April 2023, Q.Y. Liu, (HSAUP 3709-1, new host and locality record), living cultures SAUCC 3709-1.

##### GeneBank numbers.

SAUCC 3709-1: ITS = PQ351213, LSU = PQ351429, *RPB2* = PV420885, *TEF1*-*α* = PV420892.

##### Notes.

Phylogenetic analysis showed that our isolate (SAUCC 3709-1) was closely related to *Beltraniella jiangxiensis* (CGMCC 3.23486 and LC15868) (Fig. 1). There are no nucleotide position differences between our isolate (SAUCC 3709-1) and *Be. jiangxiensis* (CGMCC 3.23486 and LC15868). Morphologically, our isolate (SAUCC 3709-1) fits well with the description of *Beltraniella jiangxiensis* (CGMCC 3.23486) in having similar setae, conidiophores, conidiogenous cells, and conidia (Liu et al. 2024). Based on the high morphological similarity and no nucleotide differences, we considered our isolate (SAUCC 3709-1) as *Be. jiangxiensis*. *Be. jiangxiensis* (CGMCC 3.23486 and LC15868) were discovered on *Camellia sinensis* in Jiangxi Province, China (Liu et al. 2024). Based on morphological characteristics and phylogenetic analysis, we report our isolate (SAUCC 3709-1) as *Be. jiangxiensis*, and this study is the first report of *Be. jiangxiensis* on decaying leaves and the first recorded in Hainan Province, China.

### ﻿Dichotomous keys for Beltraniaceae

To establish a clearer taxonomic framework for distinguishing the 11 generic-level taxa within Beltraniaceae, we provide a key to the genera.

**Table:** 

1	Absence of setae	**2**
–	Presence of setae	**5**
2	Absence of separating cells	**3**
–	Presence of separating cells	**4**
3	Conidia continua	*** Parabeltrania ***
–	Conidia two celled	*** Pseudobeltrania ***
4	Conidia aseptate	*** Pseudosubramaniomyces ***
–	Conidia 3–5-septate	*** Parapleurotheciopsis ***
5	Conidiophores seta like	**6**
–	Conidiophores not seta like	**8**
6	Conidia have equatorial pores	*** Porobeltraniella ***
–	Conidia have a hyaline transverse band	**7**
7	Conidiophores have lateral branches	*** Beltraniella ***
–	Conidiophores unbranched or branched in lower part	*** Beltraniopsis ***
8	Conidiophores mostly reduced to conidiogenous cells	*** Subsessila ***
–	Conidiophores macronematous	**9**
9	Conidia without a transverse band	*** Hemibeltrania ***
–	Conidia have a hyaline transverse band	**10**
10	Conidial secession schizolytic	*** Anabeltraniomyces ***
–	Have separating cells	*** Beltrania ***

## ﻿Discussion

*Beltrania sinensis* was obtained as an endophyte from *Quercus cocciferoides* and *Fraxinus malacophylla* roots (Zheng et al. 2020). Our strain *B. sinensis* (SAUCC 5483-1) was isolated from leaves of *Quercus* species. *Beltrania sinensis* (YMF 1.05739, JS43, JS101, and SAUCC 5483-1), *B. querna* (ICMP 15825), and *Beltraniella botryospora* (TUFC 10083) were observed on *Quercus* species (Shirouzu et al. 2010; Rajeshkumar et al. 2016; Zheng et al. 2020). Beltraniaceae species appear to preferentially colonize hosts within the genus *Quercus*. Heredia et al. (2002) described *Beltraniella fertilis* as a saprobic fungus colonizing decaying leaf litter of *Mangifera indica* in Veracruz, Mexico. Magalhães et al. (2011) reported *Be. fertilis* from Brazil, where it was found associated with *Parinari alvimii*. Lin et al. (2017b) recorded the asexual morph of *Be. fertilis* (MFLUCC 17-2136, MFLUCC 17-2137, and MFLUCC 17-2138) on leaf litter from Thailand and first provided molecular data. *Beltraniella fertilis* (MFLUCC 20-0119) was discovered by Yasanthika et al. (2020) in forest soil dominated by Dipterocarpaceae in Thailand. *Beltraniella fertilis* (MFLUCC 19-0487) was discovered by Perera et al. (2020) on dried fruit of *Lithocarpus* sp. (Fagaceae) in Thailand. Our strain *Be. fertilis* (SAUCC 2153-2) was isolated from leaf litter in Yunnan Province, China. Nearly all *Be. fertilis* species act as saprophytes.

*Beltrania* and *Beltraniella* have distinct, swollen separating cells, and conidia of these two genera are turbinate or biconic, with a hyaline transverse band or several equatorial hyaline pores (Lin et al. 2017a). Traditional morphological approaches have proven inadequate for delineating boundaries between *Beltrania* and *Beltraniella*. We obtained fungal DNA sequences by amplifying the ITS and LSU regions, then reconstructed phylogenetic relationships using ML and BI analyses. In the combined ITS and LSU phylogenetic tree of Beltraniaceae (Fig. 1), species delineation showed low bootstrap support, likely due to the absence of protein-coding genes (Jeewon and Hyde 2016). For future research, we have provided the protein-coding genes *RPB2*, *TEF1*-*α* and *TUB2*, of our strains in this study.

In this study, we collected ten fungal strains from southern China. Through rigorous phylogenetic analysis and examination of morphological characteristics, we successfully identified two new species (*Beltrania veri* sp. nov. and *Beltraniella danzhouensis* sp. nov.), three new locality record species (*Beltrania sinensis*, *Beltraniella fertilis*, and *Be. jiangxiensis*), and one known species (*Be. jianfengensis*). Notably, *B. sinensis* (SAUCC 3860) and *Be. jiangxiensis* (SAUCC 3709-1) were documented as both new locality records and new host records. The results provide valuable insights into fungal taxonomy and ecological relationships, while also suggesting potential utility in plant disease management and biocontrol strategies.

## Supplementary Material

XML Treatment for Beltrania sinensis

XML Treatment for Beltrania veri

XML Treatment for Beltraniella danzhouensis

XML Treatment for Beltraniella fertilis

XML Treatment for Beltraniella jianfengensis

XML Treatment for Beltraniella jiangxiensis

## ﻿Additional information

### Use of AI

No use of AI was reported.

### Author ORCIDs

Qiyun Liu https://orcid.org/0009-0009-9545-7962

Duhua Li https://orcid.org/0009-0006-5200-2034

Zhaoxue Zhang https://orcid.org/0000-0002-4824-9716

Zhuang Li https://orcid.org/0000-0003-0583-0399

Xiuguo Zhang https://orcid.org/0000-0001-9733-8494

Jiwen Xia https://orcid.org/0000-0002-7436-7249

### Data availability

All of the data that support the findings of this study are available in the main text or Supplementary Information.

## Supplementary materials

Supplementary material 1 Supplementary figure Data type docx Explanation note (**a**) Phylogenetic tree generated from Maximum Likelihood analysis based on combined ITS and LSU sequence data of *Beltrania*. (**b**) Phylogenetic tree generated from Maximum Likelihood analysis based on combined ITS and LSU sequence data of *Beltraniella*. Bootstrap support values are shown as ML ≥ 50% first, followed by BI ≥ 0.60. *Porobeltraniella porosa* (NFCCI 3994) was used as the outgroup taxon. https://binary.pensoft.net/file/1423342This dataset is made available under the Open Database License (http://opendatacommons.org/licenses/odbl/1.0/). The Open Database License (ODbL) is a license agreement intended to allow users to freely share, modify, and use this Dataset while maintaining this same freedom for others, provided that the original source and author(s) are credited.Qiyun Liu, Duhua Li, Zhaoxue Zhang, Yuefeng Geng, Zhuang Li, Xiuguo Zhang, Jiwen Xia

Supplementary material 2 Dichotomous keys for *Beltrania* Data type docx Explanation note To distinguish species on the phylogenetic tree within *Beltrania*, we provide a key to the species. https://binary.pensoft.net/file/1423343This dataset is made available under the Open Database License (http://opendatacommons.org/licenses/odbl/1.0/). The Open Database License (ODbL) is a license agreement intended to allow users to freely share, modify, and use this Dataset while maintaining this same freedom for others, provided that the original source and author(s) are credited.Qiyun Liu, Duhua Li, Zhaoxue Zhang, Yuefeng Geng, Zhuang Li, Xiuguo Zhang, Jiwen Xia

Supplementary material 3 Dichotomous keys for *Beltraniella* Data type docx Explanation note To distinguish species on the phylogenetic tree within *Beltraniella*, we provide a key to the species. https://binary.pensoft.net/file/1423344This dataset is made available under the Open Database License (http://opendatacommons.org/licenses/odbl/1.0/). The Open Database License (ODbL) is a license agreement intended to allow users to freely share, modify, and use this Dataset while maintaining this same freedom for others, provided that the original source and author(s) are credited.Qiyun Liu, Duhua Li, Zhaoxue Zhang, Yuefeng Geng, Zhuang Li, Xiuguo Zhang, Jiwen Xia
