# Supplementary material for: ﻿Morpho-phylogenetic evidence reveals new species and records of Beltraniaceae (Amphisphaeriales, Sordariomycetes) from southern China
Source: MycoKeys. 2025 Sep 24;123:1–28. doi: 10.3897/mycokeys.123.160374 (PMC12489497; doi:10.3897/mycokeys.123.160374)
Supplement: Supplementary material 1 — Supplementary figure [file mycokeys-123-001-s001.docx]

(a)


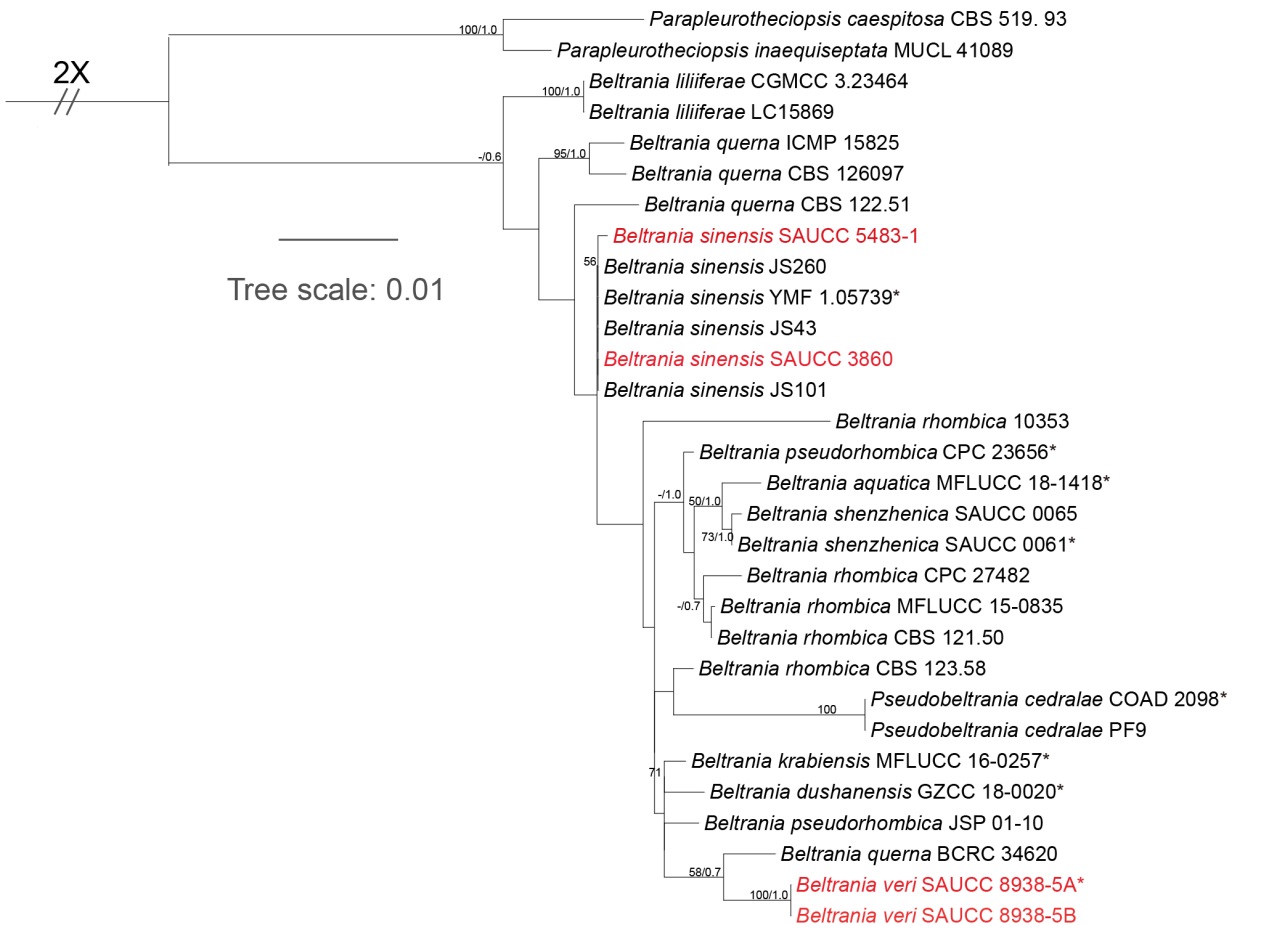


(b)


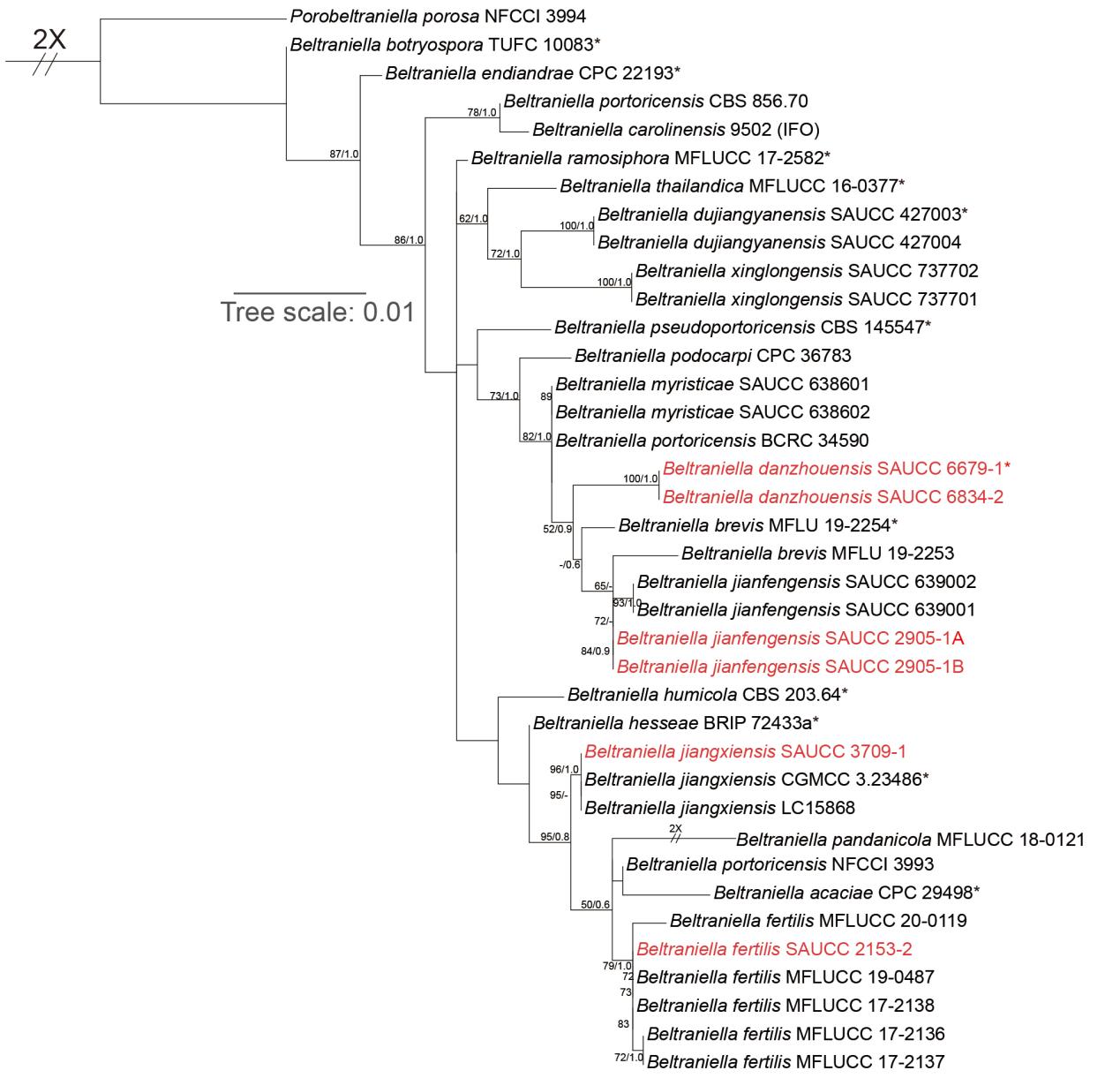


**Supplementary material 1.** **(a)** Phylogenetic tree generated from Maximum Likelihood analysis based on combined ITS and LSU sequence data of *Beltrania*. Bootstrap support values are shown as ML ≥ 50% first, followed by BI ≥ 0.60. *Parapleurotheciopsis caespitosa* (CBS 519.93) and *Parapleurotheciopsis inaequiseptata* (MUCL 41089) were used as the outgroup taxon. **(b)** Phylogenetic tree generated from Maximum Likelihood analysis based on combined ITS and LSU sequence data of *Beltraniella*. Bootstrap support values are shown as ML ≥ 50% first, followed by BI ≥ 0.60. *Porobeltraniella porosa* (NFCCI 3994) was used as the outgroup taxon.
