# Supplementary material for: ﻿Morpho-phylogenetic evidence reveals new species and records of Beltraniaceae (Amphisphaeriales, Sordariomycetes) from southern China
Source: MycoKeys. 2025 Sep 24;123:1–28. doi: 10.3897/mycokeys.123.160374 (PMC12489497; doi:10.3897/mycokeys.123.160374)
Supplement: Supplementary material 2 — Dichotomous keys for Beltrania [file mycokeys-123-001-s002.docx]

**Suppl. material 2.** **Dichotomous keys for *Beltrania***

To distinguish species on the phylogenetic tree within *Beltrania*, we provide a key to the species.

1. Conidia have one equatorial septum ........................................................ *B. aquatica*

1. Conidia are aseptate .................................................................................................. 2

2. All conidiophores > 100 µm .............................................................. *B. veri* sp. nov.

2. Not all conidiophores > 100 µm ............................................................................... 3

3. Not have separating cells ………………………………………..…….. *B. rhombica*

3. Have separating cells …………………………………………………………...…. 4

4. Some conidiophores > 300 µm ………………………………………… *B. liliiferae*

4. All conidiophores < 300 µm ……………………………….……………………… 5

5. All conidiophores ≤ 50 µm ………………………………………………………... 6

5. All conidiophores > 50 µm ……………………………………………….……….. 8

6. Separating cells smooth …………………………………………………….……... 7

6. Separating cells finely roughened ……………………………... *B. pseudorhombica*

7. Separating cells obovoid …………………………………………… *B. dushanensis*

7. Separating cells oboval …………………………………………………... *B. querna*

8. Some conidiophores septa > 10 …………………………………………. *B. sinensis*

8. All conidiophores septa < 10 ……………………………………………………… 9

9. Conidia 17–23 × 5–8 µm (including apical appendage) ………......…. *B. krabiensis*

9. Conidia 28–33 × 8.5–12 µm (including apical appendage) ……… *B. shenzhenica*
