# Supplementary material for: ﻿Morpho-phylogenetic evidence reveals new species and records of Beltraniaceae (Amphisphaeriales, Sordariomycetes) from southern China
Source: MycoKeys. 2025 Sep 24;123:1–28. doi: 10.3897/mycokeys.123.160374 (PMC12489497; doi:10.3897/mycokeys.123.160374)
Supplement: Supplementary material 3 — Dichotomous keys for Beltraniella [file mycokeys-123-001-s003.docx]

**Suppl. material 3.** **Dichotomous keys for** ***Beltraniella***

To distinguish species on the phylogenetic tree within *Beltraniella*, we provide a key to the species.

1. Setae absent ........................................................................................... *Be. humicola*

1. Setae present ............................................................................................................ 2

2. Setae septate ............................................................................................................. 3

2. Setae aseptate or not observe septate ....................................................................... 7

3. Conidia subhyaline or pale olivaceous …………………….…..…..…. *Be. fertilis*

3. Conidia hyaline or subhyaline ……………………………..…..……………….… 4

4. Conidiophores only short non-setiform .….......… *Be. danzhouensis* sp. nov.

4. Conidiophores long setiform and short non-setiform ………..…………….. 5

5. Some setae > 500 µm long ……………………………………..…. *Be. botryospora*

5. All setae < 500 µm long ……………………………………..……………………. 6

6. Separating cells ellipsoid to subglobose ………………….………… *Be. myristicae*

6. Separating cells ovoid or obovoid ………………………….…… *Be. ramosiphora*

7. Setae branched ………………………………………………..…… *Be. endiandrae*

7. Setae unbranched or not observe branched ………………………………….…… 8

8. Conidia with hyaline transverse band ...................................................................... 9

8. Conidia without hyaline transverse band or not observe ....................................... 10

9. Conidiophores very long in culture ……………………….………….... *Be. acaciae*

9. Conidiophores short ……………………………………………………... *Be. brevis*

10. Separating cells without or not observe ……………………………………….. 11

10. With separating cells ……………………………………….……………..…… 12

11. Conidiophores septate ………….……………………….…….… *Be. pandanicola*

11. Conidiophores aseptate ………………………………..……..…… *Be. thailandica*

12. Setae with large central guttules …………………………………… *Be. podocarpi*

12. Setae without large central guttules ………………………………………….…. 13

13. Separating cells limoniform ………………………………….…. *Be. portoricensis*

13. Separating cells not limoniform ……………………………………………...… 14

14. Conidia smooth ……………………………………….………………………... 15

14. Conidia verrucose ……………………………………….…………....………… 16

15. Conidiogenous cells cylindrical, polyblastic, integrating sympodially, denticulate surface, 9.2–15.3 × 2.2–5.0 μm ……….……………………….….… *Be. jianfengensis*

15. Conidiogenous cells ovoid, polyblastic, cylindrical, hyaline to subhyaline, integrated,denticulate, terminal, smooth, 6.5–9.7 × 2.8–5.4 μm …… *Be. xinglongensis*

16. Long conidiophores 113.1–259.9 × 3.1–5.8 μm and short conidiophores 13.1–31.9 × 3.2–5.7 µm................................................................................... *Be. dujiangyanensis*

16. Conidiophores 36–65 × 4–7 µm ……………….……………...…. *Be. jiangxiensis*
